# Near-Infrared Transflectance Spectroscopy Discriminates Solutions Containing Two Commercial Formulations of Botulinum Toxin Type A Diluted at Recommended Volumes for Clinical Reconstitution

**DOI:** 10.3390/bios12040216

**Published:** 2022-04-06

**Authors:** Antonio Currà, Riccardo Gasbarrone, Giuseppe Bonifazi, Silvia Serranti, Francesco Fattapposta, Carlo Trompetto, Lucio Marinelli, Paolo Missori, Eugenio Lendaro

**Affiliations:** 1 Academic Neurology Unit, Department of Medico-Surgical Sciences and Biotechnologies, Sapienza University of Rome, 04019 Terracina, Italy; 2 Research Center for Biophotonics, Sapienza University of Rome, 04100 Latina, Italy; giuseppe.bonifazi@uniroma1.it (G.B.); silvia.serranti@uniroma1.it (S.S.); 3 Ce.R.S.I.Te.S.—Research and Service Center for Sustainable Technological Innovation, Sapienza University of Rome, 04100 Latina, Italy; riccardo.gasbarrone@uniroma1.it; 4 Department of Chemical Engineering, Materials & Environment, Sapienza University of Rome, 00184 Rome, Italy; 5 Neurology Unit, Policlinico Umberto I, Department of Human Neurosciences, Sapienza University of Rome, 00185 Rome, Italy; francesco.fattapposta@uniroma1.it; 6 Department of Neuroscience, Rehabilitation, Ophthalmology, Genetics, Maternal and Child Health, University of Genoa, 16132 Genova, Italy; ctrompetto@neurologia.unige.it (C.T.); lucio.marinelli@unige.it (L.M.); 7 Department of Neuroscience, Division of Neurorehabilitation, IRCCS Ospedale Policlinico San Martino, 16132 Genova, Italy; 8 Department of Neuroscience, Division of Clinical Neurophysiology, IRCCS Ospedale Policlinico San Martino, 16132 Genova, Italy; 9 Neurosurgery Unit, Policlinico Umberto I, Department of Human Neurosciences, Sapienza University of Rome, 00185 Rome, Italy; paolo.missori@uniroma1.it; 10 Pathology Unit I.C.O.T., Department of Medical-Surgical Sciences and Bio-Technologies, Sapienza University of Rome, 04100 Latina, Italy; eugenio.lendaro@uniroma1.it

**Keywords:** NIR spectroscopy, transflectance spectroscopy, botulinum neurotoxin type A, chemometrics, partial least squares-discriminant analysis

## Abstract

Botulinum neurotoxin type A (BoNT-A) is the active substance in pharmaceutical preparations widely used worldwide for the highly effective treatment of various disorders. Among the three commercial formulations of BoNT-A currently available in Italy for neurological indications, abobotulinum A toxin (Dysport^®^, Ipsen SpA, Milano, Italy) and incobotulinum A toxin (Xeomin^®^, Merz Pharma Italia srl, Milano, Italy) differ in the content of neurotoxin, non-toxic protein, and excipients. Clinical applications of BoNT-A adopt extremely diluted solutions (10^−6^ mg/mL) for injection in the target body district. Near-infrared spectroscopy (NIRS) and chemometrics allow rapid, non-invasive, and non-destructive methods for qualitative and quantitative analysis. No data are available to date on the chemometric analysis of the spectral fingerprints acquired from the diluted commercial formulations of BoNT-A. In this proof-of-concept study, we tested whether NIRS can categorize solutions of incobotulinum A toxin (lacking non-toxic proteins) and abobotulinum A toxin (containing non-toxic proteins). Distinct excipients in the two formulations were also analyzed. We acquired transmittance spectra in the visible and short-wave infrared regions (350–2500 nm) by an ASD FieldSpec 4™ Standard-Res Spectrophotoradiometer, using a submerged dip probe designed to read spectra in transflectance mode from liquid samples. After preliminary spectra pre-processing, principal component analysis was applied to characterize the spectral features of the two BoNT-A solutions and those of the various excipients diluted according to clinical standards. Partial least squares-discriminant analysis was used to implement a classification model able to discriminate the BoNT-A solutions and excipients. NIRS distinguished solutions containing distinct BoNT-A commercial formulations (abobotulinum A toxin vs. incobotulinum A toxin) diluted at recommended volumes for clinical reconstitution, distinct proteins (HSA vs. incobotulinum A toxin), very diluted solutions of simple sugars (lactose vs. sucrose), and saline or water. Predictive models of botulinum toxin formulations were also performed with the highest precision and accuracy.

## 1. Introduction

The neurotoxic protein botulinum toxin is a metabolic product from anaerobic fermentation of the bacterium *Clostridium botulinum*, and it is considered the most potent biologic toxin. Different strains of Clostridium botulinum produce seven immunologically distinct serotypes (type A–G) that consist of complexes of the neurotoxic protein (i.e., neurotoxin) with a number of non-toxic associated proteins [1]. Botulinum neurotoxin type A (BoNT-A) is the active substance in pharmaceutical preparations widely used worldwide for the highly effective treatment of ophthalmologic, neurologic, dermatologic, gastro-enterologic, urologic, and many other specialist disorders [2].

Today, among the different formulations of BoNT-A currently available in Italy for neurological indications, there are abobotulinum A toxin (Dysport^®^, Ipsen SpA, Milano, Italy) and incobotulinum A toxin (Xeomin^®^, Merz Pharma Italia srl, Milano, Italy). These formulations differ in the content of neurotoxin, non-toxic protein, and excipients [3]. For a safe and effective treatment, each summary of product characteristics recommends specific volume dispersion of vials delivered in licensed indications. This volume is 2 mL unpreserved saline for a 100-unit vial of incobotulinum A and onabotulinum A toxins, and 2.5 mL unpreserved saline for a 500-unit vial of abobotulinum A toxin. The resulting “clinical dilutions” (i.e., those present in the syringes for injections) are 50 units/mL for inco- and onabotulinum A toxins, and 200 units/mL for abobotulinum A toxin. Owing to the estimated lethal dose for botulinum toxin of approximately 0.09 to 0.15 µg intravenously or intramuscularly [4], these therapeutic preparations are extremely diluted volumes, approximately in the order of 10^−6^ mg/dL.

Spectroscopic techniques have been widely used in experimental studies aiming to explore the pharmacological [5], biochemical [6], and molecular [7] aspect of botulinum toxins, and, more recently, to develop point-of-care assays able to detect very low toxin concentrations [8]. The optical properties of a sample describe the interaction of an examined material with light, and specifically, the energy transfer between the electromagnetic radiation and matter. Near-infrared spectroscopy (NIRS) is a simple, quick, nondestructive technique that provides multi-constituent analysis on virtually any matrix, including liquid chemical formulations, with levels of accuracy and precision that are comparable to primary reference methods [9]. The near-infrared (NIR) region (780–2500 nm) of the electromagnetic radiation is situated between the red band of the visible light and the mid infrared region. The NIR signal—i.e., the spectrum—results from the absorbance of light due to molecular vibrations of hydrogen bonds, such as C-H, N-H, O-H, and therefore contains chemical and physical information on the sample and its constituents. NIR spectra exhibit broad overlapping NIR absorption bands that require special mathematical procedures for data analysis (i.e., chemometrics) [10]. As the calibration and validation of the measured NIR spectral data are correlated through statistical methods to reference data, NIR spectroscopy is considered a secondary analytical method.

NIRS applications in the pharmaceutical analysis range from the identification of raw materials to the fully on-line quality control of the finished and packed pharmaceutical end products [11]. Because concentrations of 5000 ppm (mgL^−1^) or 0.5% (*w*/*v*) are roughly regarded as a common limit of quantification for NIRS [12], this technique is not used to analyze the active principles in the final-use conditions of standard clinical practice. To our knowledge, NIRS has never been applied to analyze diluted formulations of BoNT-A, nor are data available on the chemometric analysis of spectral fingerprints acquired from samples of highly diluted solutions of BoNT-A. Developing a procedure that can quickly reveal the presence of the neurotoxin at similar limits of detection is desirable to improve the existing methods while eliminating the need for living animals (toxin biological activity is determined as LD50 in the mouse). Botulinum toxin formulations differ in terms of neurotoxin unit content, total clostridial protein content (due to the presence or absence of complexing proteins), and neurotoxin protein load. Pharmaceutical technology also requires the adoption of different types or quantity of excipients (lactose vs. sucrose, low or high albumin content), as an expression of the different production processes. Before injection in the target structure (i.e., muscles, glands, cutis, etc.), both formulations are reconstituted by using unpreserved saline at the “clinical dilutions“ described beforehand. Therefore, in this proof-of-concept study, we recorded the NIR transmittance spectra from several liquid formulations, including two commercial botulinum toxin vials prepared at “clinical dilution”, separate volumes of each single excipient present in the commercial vials prepared at the same dilution, and volumes of the unpreserved saline routinely used for diluting the toxin vials.

## 2. Materials and Methods

### 2.1. Preparation and Dilutions of the Samples: Commercially Available Formulations of Botulinum Toxin, Excipients, Saline, and Water

Separate solutions of abobotulinum A toxin (Dysport, Ipsen, France), incobotulinum A toxin (Xeomin, Merz GmBh, Germany), excipients of abobotulinum A toxin (human serum albumin (HSA) and lactose), excipients of incobotulinum A toxin (HSA and sucrose), HSA, lactose, sucrose, saline, and water were prepared (Table 1).

The components of the two commercial formulations of botulinum toxin are shown in Table 2.

The commercial formulations were diluted according to the standard used in clinical practice: abobotulinum A toxin at 500 units in 2.5 mL (200 U/mL) and incobotulinum A toxin at 100 units in 2 mL (50 U/mL), using saline.

The solutions of each excipient and solvent were prepared at the same dilutions as for the toxin formulations. We used HSA (Albital 200 g/L, Kedrion Biopharma, Barga, LU, Italy), sucrose, and lactose of analytical grade (Sigma-Aldrich, Milan, Italy), saline (Fresenius Kabi Italia, Isola della Scala, VR, Italy). Ultrapure water was produced by Milli-Q^®^ system (Merck-Millipore, Burlington, MA, USA).

Just before proceeding to spectra acquisition, the temperature and the potential of hydrogen (pH) of the solutions were measured by using a Hi 255 Combined meter (HANNA instruments^®^, Woonsocket, RI, USA) and a digital instant read thermometer (Thermometer H-B Instrument™ Easy-Read™, Thermo Fisher Scientific Instruments, Waltham, MA, USA).

### 2.2. Spectra Acquisition and Data Handling

We used a portable spectrophotoradiometer system (FieldSpec 4™ Standard-Res Spectrophotoradiometer; ASD Inc., Boulder, CO, USA) to acquire transmittance spectra in the Vis-SWIR regions by means of a dip probe for performing liquid measurements in transflectance mode. Spectra acquisition was performed in controlled environmental condition.

The transflectance probe fiber (2 m length, 600 μm LOH S/S, 0.22 NA) is submerged into the liquid sample, which enters a cavity in the probe through a slit at the tip. The cavity is equipped with an optically transparent window placed at the distal end of the fiber and a small mirror placed at the bottom of the cavity.

The ASD FieldSpec 4^®^ has a resolution equal to 3 nm and 10 nm, at 700 nm and 1400/2000 nm, respectively [13]. Spectral device sensing architecture consists of 3 different, and separated, holographic diffraction gratings, each with a separate detector (VNIR detector 350–1000 nm, SWIR 1 detector 1001–1800 nm, SWIR 2 detector 1801–2500 nm).

**Figure 1:**
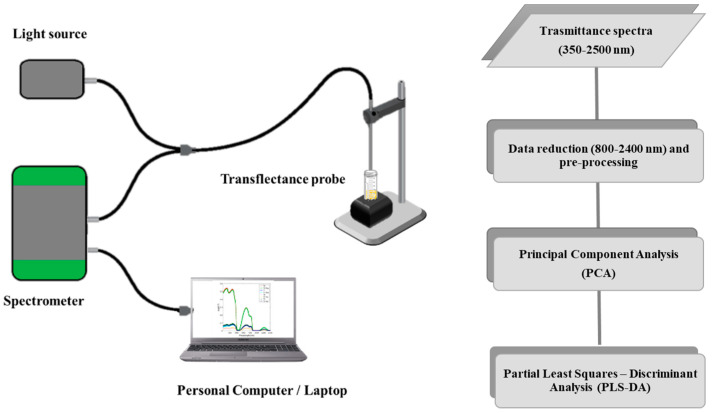
Schematic representations of the utilized spectrophotometer (**left**) and the experimental set-up (**right**).

A schematic representation of the instrument set-up and experimental design is shown in Figure 1.

A total of 50 spectra were collected from each solution (*n* = 9) separately (total 450 spectra) in a 15 mL Falcon tube accommodated in a black plastic support designed for the purpose.

Data acquisition and calibration procedures were performed using ASD RS3 software [13]. The calibration of the spectroradiometer was performed with the cavity of the dip probe empty by collecting the reflected light of the mirror as a “white” reference. This procedure allowed collecting spectra on the transparent liquid also, i.e., ultrapure water. The calibration was repeated before acquisition from each sample.

Spectra “.asd” data files were stacked as transmittance spectra into an ASCII text file using ViewSpec Pro (Ver. 6.2.0; ASD Inc., Boulder, CO, USA), then imported into MATLAB^®^ (R2019a, Ver. 9.6.0; The Mathworks, Inc., Natick, MA, USA) using an ad hoc routine. Data were stored into DataSet Objects (DSOs) and analyzed using the PLS_toolbox (Ver. 8.2.1; Eigenvector Research, Inc., Wenatchee, WA, USA).

To exclude the visible region from analysis, all processing was developed with reference to 800–2400 nm spectral range (NIR-SWIR region).

### 2.3. Spectra Pre–Processing and Exploratory Analysis

Standard normal variate algorithm was used for reducing scattering phenomena that affect the illuminant or detectors [14,15]. To enhance signal/noise ratio and remove high-frequency components of noise, Savitzky–Golay smoothing with a 33-point window was used [16]. Data were then subjected to the mean center algorithm, which centers the columns to have zero mean.

An exploratory analysis of decomposed spectra data was performed by using principal component analysis (PCA), which extracts the dominant patterns of the spectra data matrix in terms of the product of two smaller matrices of scores and loadings [17]. Principal components (PCs) were chosen by exploring the eigenvalues plot. Outliers and not informative data were identified and excluded.

First, we aimed to see whether NIR transflectance spectroscopy could distinguish very diluted solutions containing distinct combinations of proteins, sugars, and salt (PCA i, incobotulinum A toxin (inco100alb1sac46), equiactive abobotulinum A toxin (abo400alb01lat2), albumin (alb1), sucrose (sac46), lactose (lact1), saline (NC09), and water (AD)).

Second, we sought to discriminate the solutions of commercial toxin formulations at standard clinical dilutions that differed in the content of toxin-related proteins (PCA ii, incobotulinum A toxin (inco100alb1sac46), abobotulinum A toxin at standard clinical dilutions (abo100alb0025lat05), and their excipients (Eccinco1, EccAbo)).

Third, we performed the same analysis on solutions diluted at volumes containing neurotoxin proteins of equivalent biologic activity (PCA iii, incobotulinum A toxin (inco100alb1sac46), equiactive abobotulinum A toxin (abo400alb01lat2), and their excipients (Eccinco1, EccAbo)).

Fourth, we tested diluted solutions of each toxin formulation with solutions of the respective excipients (PCA iv, abobotulinum A toxin at standard clinical dilutions (abo100alb0025lat05), and its excipients (EccAbo); and PCA v., incobotulinum A toxin (inco100alb1sac46), and its excipients (Eccinco1)).

Fifth, we contrasted the two solutions of commercial formulations diluted at volumes containing neurotoxin proteins of presumed equivalent biologic activity (PCA vi, incobotulinum A toxin (inco100alb1sac46), equiactive abobotulinum A toxin (abo400alb01lat2)).

Sixth, we contrasted the two solutions of commercial formulations at standard clinical dilutions (PCA vii, incobotulinum A toxin (inco100alb1sac46), abobotulinum A toxin at standard clinical dilutions (abo100alb0025lat05)).

Seventh, we contrasted the two solutions with the same amount of one protein (i.e., HSA) but different content of other proteins (i.e., incobotulinum A toxin) and sugar (PCA viii, incobotulinum A toxin (inco100alb1sac46), and equidiluted HSA (alb1)).

Finally, we contrasted solutions of distinct sugars (lactose and sucrose) diluted at the same volumes used for reconstituting the toxin formulations (PCA ix, Lactose (lact1), and sucrose (sac46)) and solutions of saline and water (PCA x, saline (NC09), and water (AD)).

### 2.4. Classification Models

Partial least square-discriminant analysis (PLS-DA) is a supervised technique for pattern recognition that utilizes the partial least square regression to develop a model able to predict the class number for each sample under study [18,19]. We used PLS-DA to discriminate incobotulinum A toxin and abobotulinum A toxin spectral signatures in the spectral range 800–2400 nm.

The first classification model was set up by randomly splitting the whole dataset into two parts by using the Kennard/Stone algorithm. A total of 70% of the data were used as a calibration set, the remaining 30% as a test set. Venetian Blinds were used as a cross-validation method for assessing the optimal complexity of the models and for choosing the number of Latent variables (LVs; n = 4).

A second classification model was set up by using spectral values at wavelengths chosen by evaluating the variable importance in projection (VIP) scores of the first PLS-DA. The VIP scores are a metric of the importance of each variable in the projection used in a PLS model [20]. VIP scores < 1 are considered less important and were excluded from the model. This model was calibrated, cross-validated, and validated with the same approach used for the preceding models, using, in this case, 3 LVs.

The confusion matrix was considered for evaluating the classifiers’ performance and for calculating performance metrics, i.e., precision (ratio of correctly predicted positive observations to the total predicted positive observations); accuracy (ratio of correctly predicted observations to the total observations); sensitivity (correctly recognized samples belonging to a determined class); specificity (correctly rejected samples belonging to all other classes) [21].

## 3. Results

### 3.1. Exploratory Analysis

**Figure 2:**
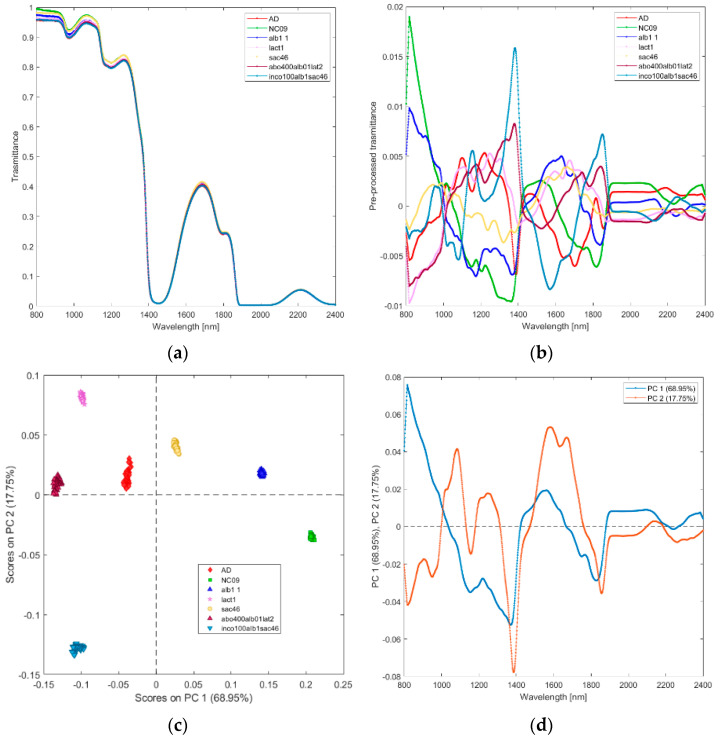
Average raw (**a**) and pre-processed (**b**) transmittance spectra for solutions of incobotulinum A toxin (inco100alb1sac46), abobotulinum A toxin (abo400alb01lat2), HSA (alb1), sucrose (sacc46), lactose (lact1), saline (NaC09), and water (AD). PC1−PC2 score plot (**c**) and loading plot of PC1 and PC2 (**d**) for incobotulinum A toxin (inco100alb1sac46), equiactive abobotulinum A toxin (abo400alb01lat2), HSA (alb1), sucrose (sacc46), lactose (lact1), saline (NaC09), and water (AD).

**(i) Solutions of incobotulinum A toxin (inco100alb1sac46), equiactive abobotulinum A toxin (abo400alb01lat2), albumin (alb1), sucrose (sac46), lactose (lact1), saline (NC09), and water (AD).** The average raw and pre–processed spectra of incobotulinum A toxin, abobotulinum A toxin, albumin, sucrose, lactose, saline, and water are shown in Figure 2a,b, respectively. PCA score plot and loadings plot of the first two principal components are shown in Figure 2c,d. PC1 captured 69% of the total variance, while PC2 added a further 18% (Figure 2c). The solutions scores are in distinct quadrants. Sucrose and albumin solutions scores are in the positive quadrants of PC1 and PC2 due to the wavelengths around 1450–1650 nm (corresponding to the first overtone of OH and NH and to the st overtone combinations of CH, as shown in Figure 2d). Water, lactose, and abobutulinum A toxin solutions scores are in the second quadrant of the PCA scores plot due to the wavelength ranges around 1000–1150 nm (corresponding to RNH_2_, the second overtone of NH and the second overtone of CH), 1200–1350 nm (C-H stretching), 1700–1750 nm (corresponding to the first overtone of CH and to the first overtone of SH). Incobotulinum A toxin and saline solution scores are in the negative quadrant of PC2 due to the wavelength ranges 800–1000 nm (corresponding to the third overtone of CH, third overtone of NH, and second overtone of OH), 1350–1500 nm (corresponding to RNH_2_, the first overtone of OH and first overtone combinations of CH) and 1750–2100 nm (SH, CONH_2_, OH first overtone and NH + OH combination bands regions). PC2 discriminates unequivocally incobotulinum A toxin (negative quadrant) from abobutulinum A toxin (positive quadrant).

**Figure 3:**
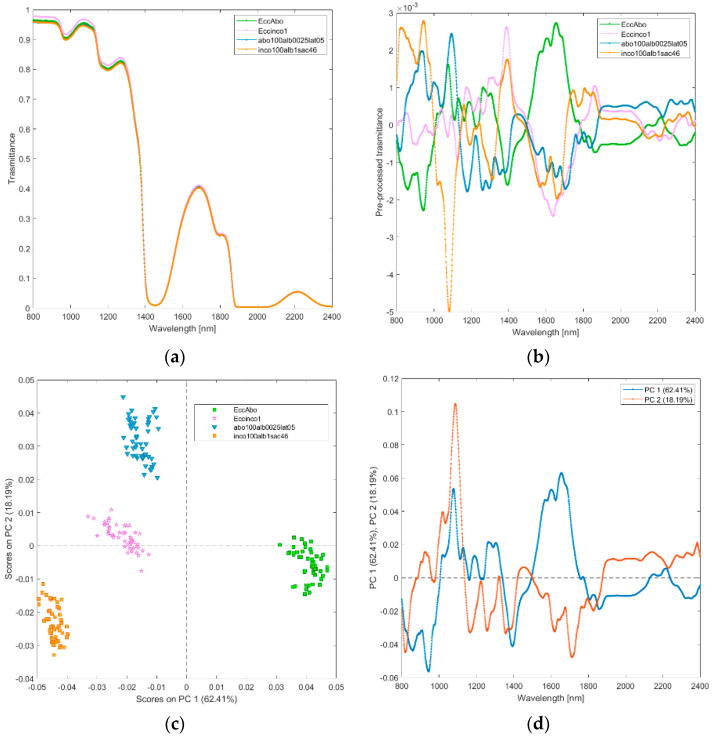
Average raw (**a**) and pre-processed (**b**) transmittance spectra for incobotulinum A toxin (inco100alb1sac46) and abobotulinum A toxin (abo100alb0025lat05), and their excipients (EccInco, EccAbo). PC1−PC2 score plot (**c**) and loading plot of PC1 and PC2 (**d**) for incobotulinum A toxin (inco100alb1sac46), abobotulinum A toxin (abo100alb0025lat05), and their excipients (EccInco, EccAbo).

**(ii) Solutions of incobotulinum A toxin (inco100alb1sac46), abobotulinum A toxin at standard clinical dilutions (abo100alb0025lat05), and their excipients (Eccinco1, EccAbo).** The raw and pre-processed transmittance spectra of incobotulinum A toxin, abobotulinum A toxin at standard clinical dilutions, and their excipients are shown in Figure 3a,b. PCA score plot and loadings plot of the first two principal components are shown in Figure 3c,d. PC1 captured the 62% of the total variance, while PC2 added a further 18% and PC3 another 12%. The scores of the two botulinum toxin A solutions are clearly separated by PC2 (Figure 3c); those of the excipients are separated by PC1. The scores of the abobotulinum A toxin at standard clinical dilutions are in the negative space of PC1 and positive space of the PC2, while incobotulinum A toxin scores are in the negative space of PC1 and PC2 due to the wavelength ranges around 800–900 nm (corresponding to RNH_2_, and the third overtone of NH; Figure 3d), 1350–1450 nm (CH, CONH_2_, first overtone of OH and first overtone combinations of CH) and 1750–1850 nm (SH, first overtone of SH and first overtone of CH). Incobotulinum A toxin excipients solution scores are in the negative space of PC1, while the excipients solution scores of abobotulinum toxin A are in the positive space of PC1. PC2 separates the two solutions of toxins and their excipients, while PC1 allocates the solution excipients in two distinct clusters.

**Figure 4:**
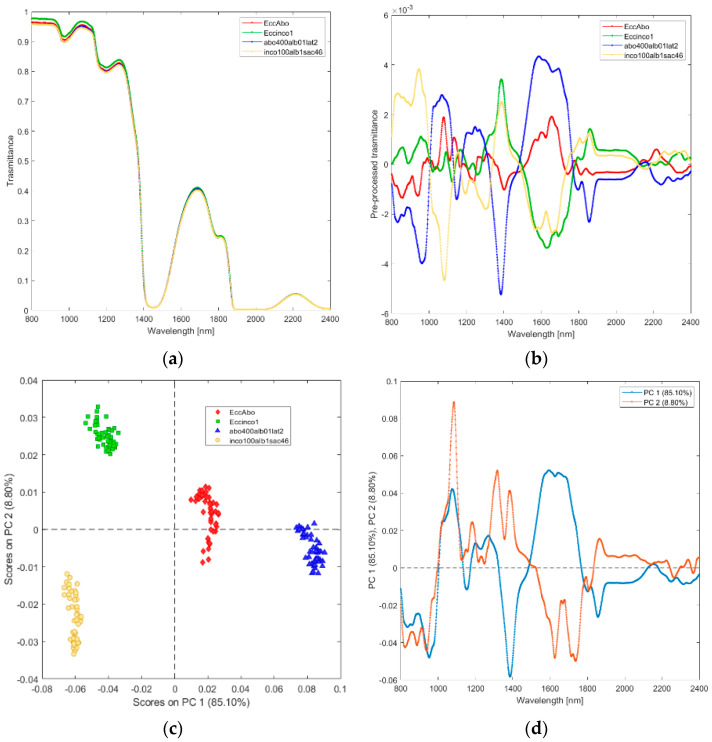
Average raw (**a**) and pre-processed (**b**) transmittance spectra for incobotulinum A toxin (inco100alb1sac46) and equiactive abobotulinum A toxin (abo400alb01lat2), and their excipients (EccInco, EccAbo). PC1−PC2 score plot (**c**) and loading plot of PC1 and PC2 (**d**) for incobotulinum A toxin (inco100alb1sac46), equiactive abobotulinum A toxin (abo400alb01lat2), and their excipients (EccInco, EccAbo).

**(iii) Solutions of incobotulinum A toxin (inco100alb1sac46), equiactive abobotulinum A toxin (abo400alb01lat2), and their excipients (Eccinco1, EccAbo).** The raw and pre-processed transmittance spectra of incobotulinum A toxin, abobotulinum A toxin, and their excipients are shown in Figure 4a,b. PCA score plot and loadings plot of the first two principal components are shown in Figure 4c,d. PC1 captured 85% of the total variance, while PC2 captured 9%. Scores of the two botulinum toxin A solutions are clearly separated by PC1 scores (Figure 4c). The scores of the solutions of abobotulinum A toxin and its excipients are in the positive space on PC1 due to the wavelength ranges around 1000–1150 nm (corresponding to RNH_2_, the second overtone of NH and the second overtone of CH, Figure 4d), 1200–1350 nm (C-H stretching) and 1500–1800 nm (corresponding to RNH_2_, SH, the first overtone of NH, first overtone of CH and first overtone of SH). The scores of incobotulinum A toxin and its excipients are in the negative space on PC1 due to the wavelength ranges around 800–1000 nm (corresponding to RNH_2_, the second overtone of OH, the third overtone of NH; the third overtone of CH), 1150–1200 nm (C-H stretching and CH second overtone), 1350–1500 nm (corresponding to the first overtone of OH, first overtone combinations of CH and first overtone of NH), 1800–2400 nm (CONH_2_, second overtone CO, NH + OH and CH + CH combination bands regions).

**Figure 5:**
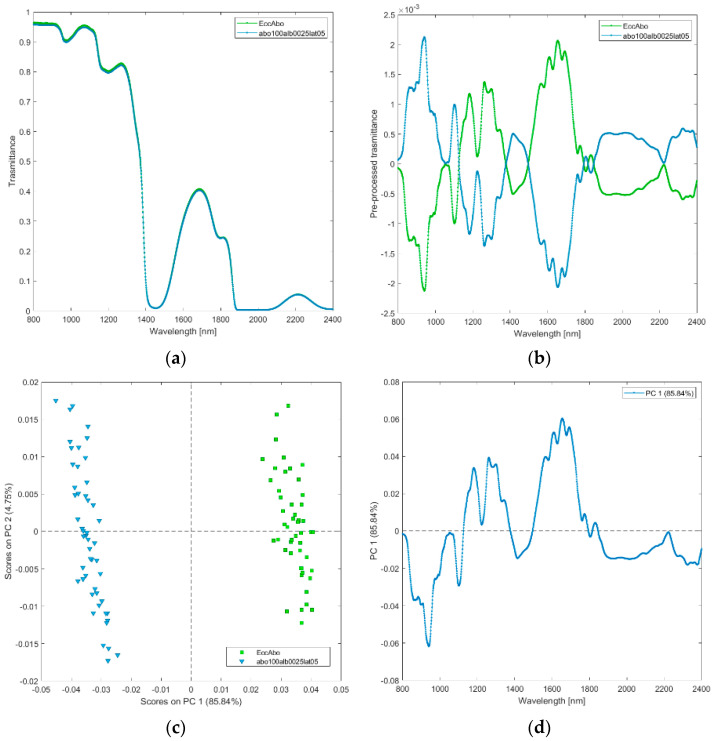
Average raw (**a**) and pre-processed (**b**) transmittance spectra for abobotulinum A toxin at standard clinical dilutions (abo100alb0025lat05), and its excipients (EccAbo). PC1−PC2 score plot (**c**) and loading plot of PC1 (**d**) for abobotulinum A toxin at standard clinical dilutions (abo100alb0025lat05), and its excipients (EccAbo).

**(iv) Solutions of abobotulinum A toxin at standard clinical dilutions (abo100alb0025lat05), and its excipients (EccAbo).** The raw and pre-processed transmittance spectra of abobotulinum A toxin and its excipients are shown in Figure 5a,b. PCA scores plot and loadings plot are shown in Figure 5c,d. Abobotulinum A toxin is discriminated from its excipients by the relative scores on PC1, which captured 86% of the total variance (Figure 5c). The differences are explained by the loadings plot (Figure 5d). Abobotulinum A toxin scores are in the PC1 negative space due to the wavelength ranges around 800–1150 nm (corresponding to RNH_2_, the second overtone of OH, the third overtone of NH; the third overtone of CH; Figure 5d), 1350–1500 nm (CH, CONH_2_, the first overtone of OH, the first overtone NH, the first overtone of OH, the first overtone combinations of CH), and 1800–2400 nm (CONH_2_, second overtone CO, NH + OH and CH + CH combination bands regions).

**Figure 6:**
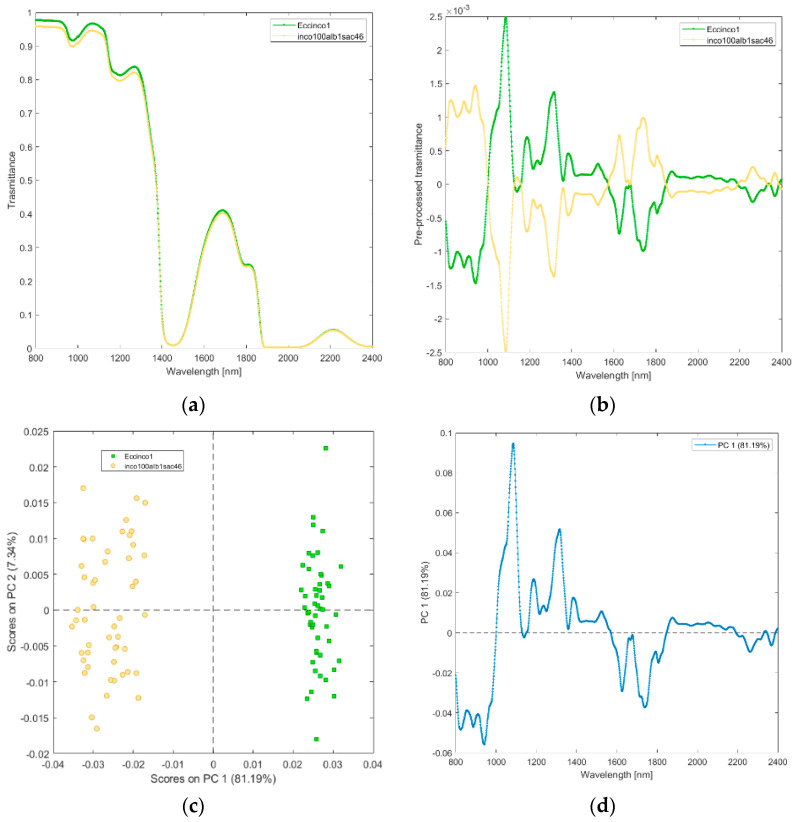
Average raw (**a**) and pre-processed (**b**) transmittance spectra for incobotulinum A toxin (inco100alb1sac46), and its excipients (EccInco). PC1−PC2 score plot (**c**) and loading plot of PC1 (**d**) for incobotulinum A toxin (inco100alb1sac46), and its excipients (EccInco).

**(v) Solutions of incobotulinum A toxin (inco100alb1sac46), and its excipients (Eccinco1).** The raw and pre-processed transmittance spectra of incobotulinum A toxin and its excipients are shown in Figure 6a,b. PCA scores plot and loadings plot are shown in Figure 6c,d. PC1 captured 81% of the total variance and discriminated incobotulinum A toxin from its excipients (Figure 6c). Incobotulinum A toxin scores are in the PC1 negative space mainly due to the wavelength ranges 800–1000 nm (corresponding to RNH_2_, the second overtone of OH, the third overtone of NH; the third overtone of CH; Figure 6d), 1600–1850 nm (corresponding to RNH_2_, SH, the first overtone of NH, first overtone of CH and first overtone of SH). Excipients are in the PC1 positive space.

**Figure 7:**
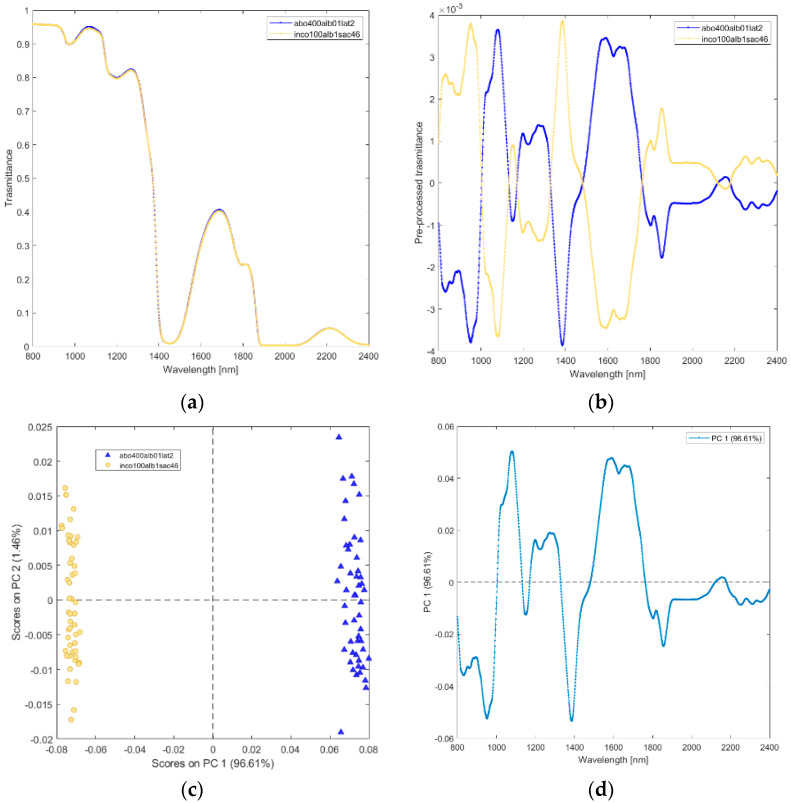
Average raw (**a**) and pre-processed (**b**) transmittance spectra for incobotulinum A toxin (inco100alb1sac46), and equiactive abobotulinum A toxin (abo400alb01lat2). PC1−PC2 score plot (**c**) and loading plot of PC1 (**d**) for incobotulinum A toxin (inco100alb1sac46), and equiactive Abobotulinum A toxin (abo400alb01lat2).

**(vi) Solutions of incobotulinum A toxin (inco100alb1sac46_4_), equiactive abobotulinum A toxin (abo400alb01lat2).** The raw and pre-processed transmittance spectra of incobotulinum A toxin and its excipients are shown in Figure 7a,b. PCA scores plot and loadings plot are shown in Figure 7c,d. PC1 captured 97% of the total variance (Figure 7c). The scores of incobotulinum A toxin are in PC1 negative space due to the wavelength ranges around 800–1000 nm (corresponding to RNH_2_, the second overtone of OH, the third overtone of NH; the third overtone of CH; Figure 7d), 1150–1180 nm (O-H stretching for water), 1350–1500 nm (corresponding to CH, CONH_2_, the first overtone of OH, the first overtone NH, the first overtone of OH, the first overtone combinations of CH) and 1780–2400 nm (CONH_2_, second overtone CO, NH + OH and CH + CH combination bands regions). The scores of abobutolinum A toxin are found in the PC1 positive space.

**Figure 8:**
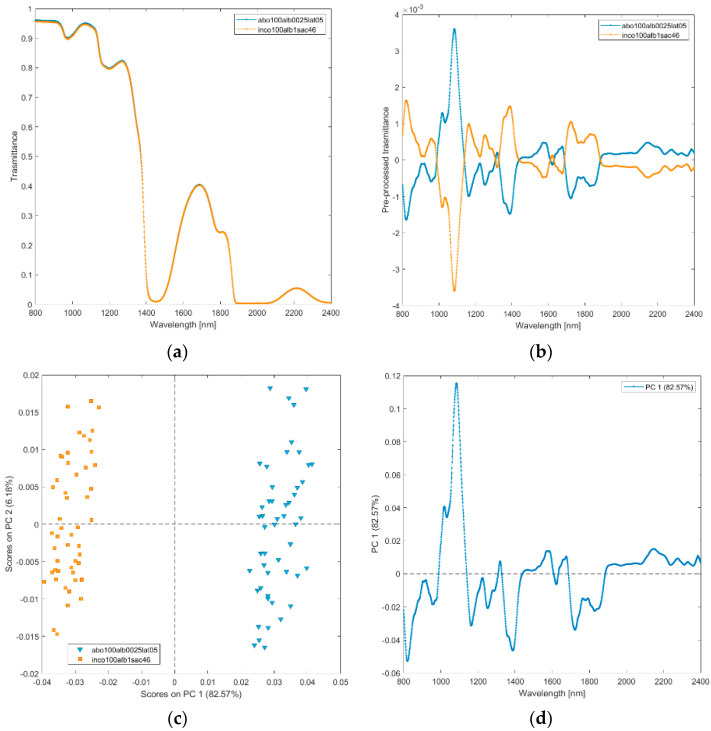
Average raw (**a**) and pre-processed (**b**) transmittance spectra for incobotulinum A toxin (inco100alb1sac46) and abobotulinum A toxin (abo100alb0025lat05). PC1−PC2 score plot (**c**) and loading plot of PC1 (**d**) for incobotulinum A toxin (inco100alb1sac46), abobotulinum A toxin (abo100alb0025lat05).

**(vii) Incobotulinum A toxin (inco100alb1sac46), abobotulinum A toxin at standard clinical dilutions (abo100alb0025lat05).** The raw and pre-processed transmittance spectra of incobotulinum A toxin, and abobotulinum A toxin at standard clinical dilutions are shown in Figure 8a,b. PCA score plot and loadings plot are shown in Figure 8c,d. PC1 captured 83% of the total variance, while PC2 captured 6%. Scores of the two botulinum toxin A solutions are sharply separated by PC1 scores (Figure 8c). The scores of the solutions of abobotulinum A toxin are in the positive space on PC1 due to the wavelength ranges around 1000–1150 nm (corresponding to RNH_2_, the second overtone of OH, the third overtone of NH; the third overtone of CH; Figure 8d), 1300–1350 nm (first overtone combinations of CH), 1450–1550 nm (corresponding to H_2_O, the first overtone of OH, the first overtone combinations of CH and first overtone of NH), 1650 nm (first overtone of CH), 1900–2400 nm (CONH_2_, second overtone CO, NH + OH and CH + CH combination bands regions).

**Figure 9:**
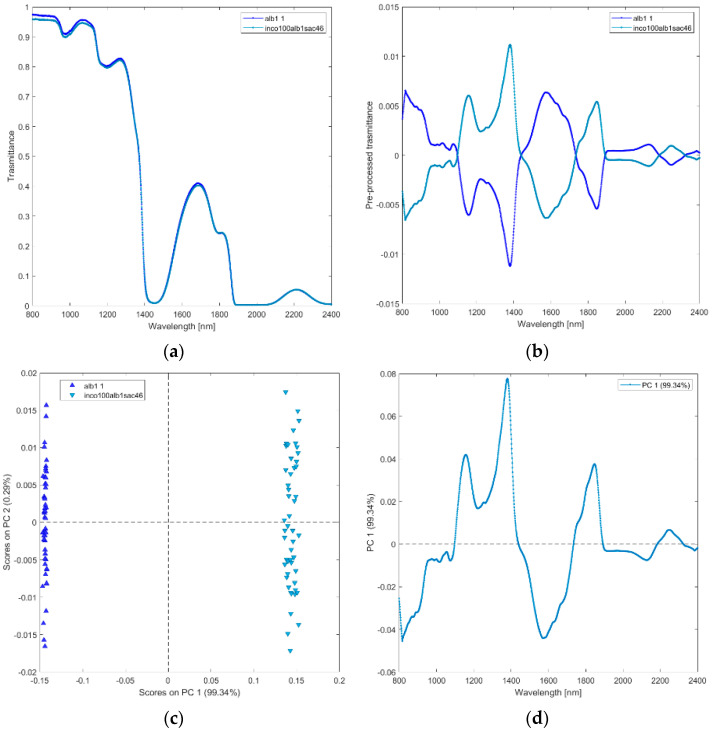
Average raw (**a**) and pre-processed (**b**) transmittance spectra for incobotulinum A toxin (inco100alb1sac46), and HSA (alb1). Scores on PC1 vs. PC2 (**c**) and loading plot of PC1 (**d**) for incobotulinum A toxin (inco100alb1sac46), and HSA (alb1).

**(viii) Solutions of incobotulinum A toxin (inco100alb1sac46), and equidiluted HSA (alb1).** The raw and pre-processed transmittance spectra of incobotulinum A toxin and HSA are shown in Figure 9a,b. PCA scores plot and loadings plot are shown in Figure 9c,d. PC1 captured 99% of the total variance. The scores of incobotulinum A toxin are in the negative space of PC 1 due to the wavelength ranges around 800–1100 nm(corresponding to RNH_2_, the second overtone of OH, the third overtone of NH; the third overtone of CH; Figure 9d), 1450–1750 nm (corresponding to RNH_2_, SH, the first overtone of NH, first overtone of CH and first overtone of SH), and 1900–2200 nm (CONH_2_, second overtone CO, NH + OH and CH + CH combination bands regions). The scores of HSA are clustered in the positive space of PC 1.

**Figure 10:**
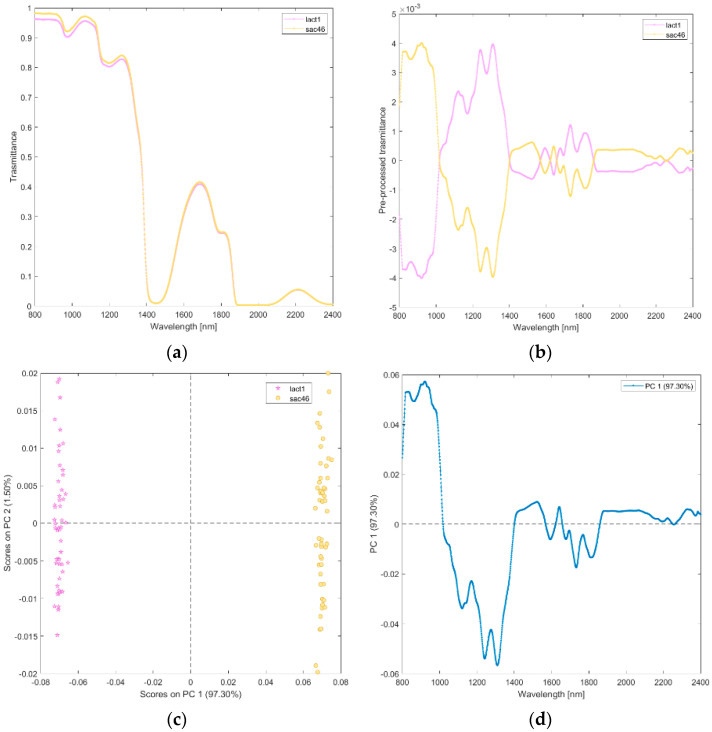
Average raw (**a**) and pre-processed (**b**) transmittance spectra for sucrose (sacc46), and lactose (lact1). PC1−PC2 score plot (**c**) and loading plot of PC1 (**d**) for sucrose (sacc46), and lactose (lact1).

**(ix) Solutions of lactose (lact1), and sucrose (sac46).** The raw and pre-processed transmittance spectra of lactose and sucrose are shown in Figure 10a,b. PCA scores plot and loadings plot of the first principal components are shown in Figure 10c,d. PC1 captured 97% of the total variance. As shown in Figure 10c, the scores of lactose are in the negative space of PC 1 due to the wavelength ranges around 1000–1450 nm (mainly corresponding to water region associated with O-H stretching, the second overtone of CH and the first overtone combinations of CH; Figure 10d) and 1550–1850 nm (corresponding to CH, and the first overtone of CH), while the scores of sucrose are clustered in the positive space of PC 1.

**Figure 11:**
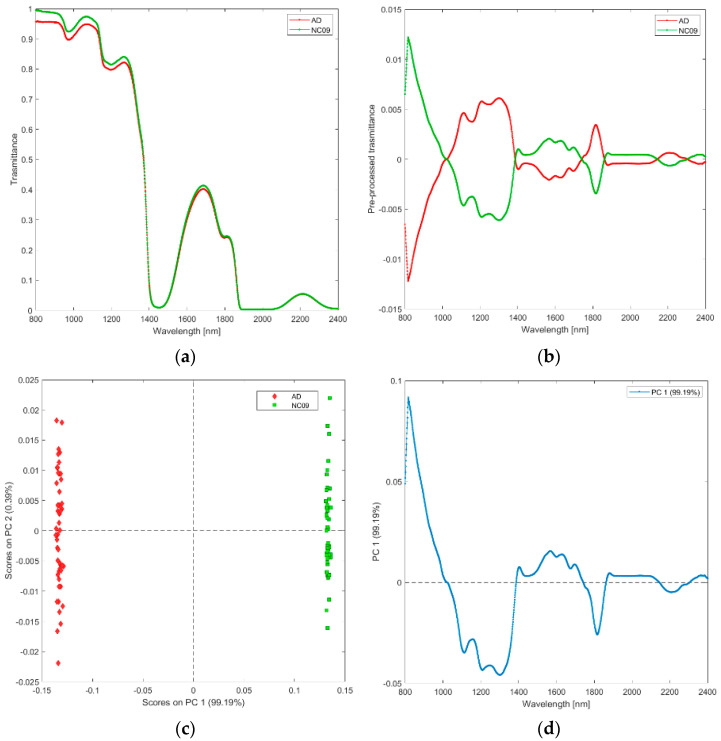
Average raw (**a**) and pre-processed (**b**) transmittance spectra for saline (NaC09), and water (AD). PC1−PC2 score plot (**c**) and loading plot of PC1 (**d**) for saline (NaC09), and water (AD).

**(x) Solutions of Saline (NC09), and water (AD).** The raw and pre-processed transmittance spectra of saline and ultrapure water are shown in Figure 11a,b. PCA scores plot and loadings plot of the first principal components are shown in Figure 11c,d.

PC1 captured 99% of the total variance. As shown in Figure 11c, the scores of water are in the negative space of PC 1 due to the wavelength ranges around 800–1000 nm (corresponding to water region associated with O-H stretching and second overtone of OH; Figure 11d) and 1750–1850 nm (corresponding to the water region associated with O-H stretching), while the scores of saline are clustered in the positive space of PC 1.

### 3.2. Classification Models for Discriminating Solutions of Incobotulinum A Toxin and Abobotulinum A Toxin

The PLS-DA performance metrics using the wavelength range 800–2400 nm discriminated the solutions of incobotulinum A toxin from those of abobotulinum A toxin, as reported in Table 3, showing that the values of Sensitivity, Specificity, Precision, and Accuracy are all proximal to the unit during calibration, cross-validation, and validation phases of the model.

**Figure 12:**
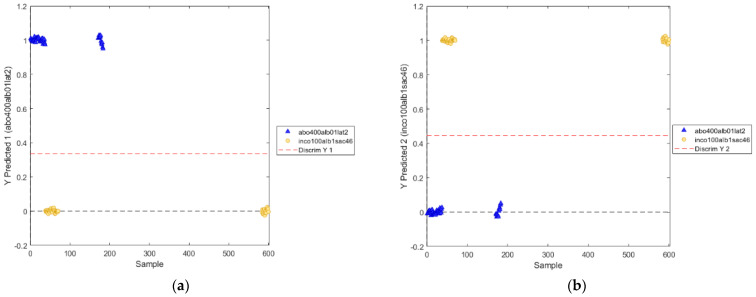
Positions of the discrimination boundary as determined by PLS−DA model (utilized wavelength range: 800−2400 nm) for the two classes: abobotulinum A toxin (**a**) and incobotulinum A toxin (**b**).

The positions of the discrimination boundary for the two modeled classes are shown in Figure 12.

**Figure 13:**
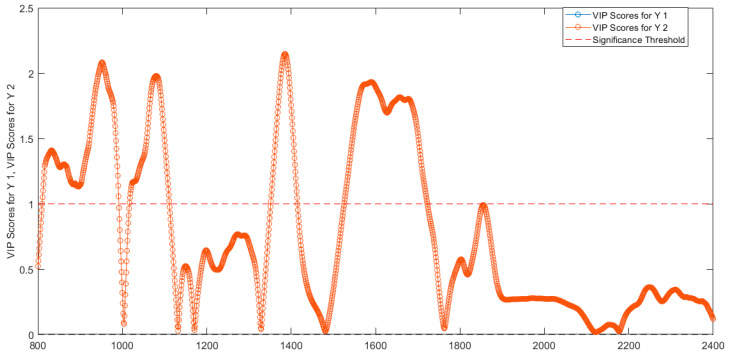
VIP scores determined from PLS−DA model for the two classes of abobotulinum A toxin (VIP scores for Y1) and incobotulinum A toxin (VIP scores for Y2).

By using the VIP scores > 1 to evaluate the importance of each variable in the projection used in a PLS model [20], the selected wavelengths were: 811–991 nm, 1018–1111 nm, 1352–1414 nm, 1527–1722 nm (Figure 13). The wavelengths 811–991 nm correspond to RNH_2_, the second overtone of OH, the third overtone of NH, and the third overtone of CH. The range of wavelengths around 1018–1111 nm are associated with RNH_2_, the second overtone of OH, the second and third overtone of NH). The wavelength range 1352–1414 nm corresponds to H_2_O, the first overtone of OH, the first overtone of NH, the first overtone combinations of CH. Finally, the wavelengths 1527–1722 nm correspond to RNH_2_, SH, the first overtone of NH, the first overtone of CH and first overtone of SH.

The application of the classification model centered on the significant wavelengths alone demonstrated the correct discrimination among the solutions of incobotulinum A toxin from those of abobotulinum A toxin. The model performed very well, as shown by the values of Sensitivity, Specificity, Precision, and Accuracy (Table 4).

**Figure 14:**
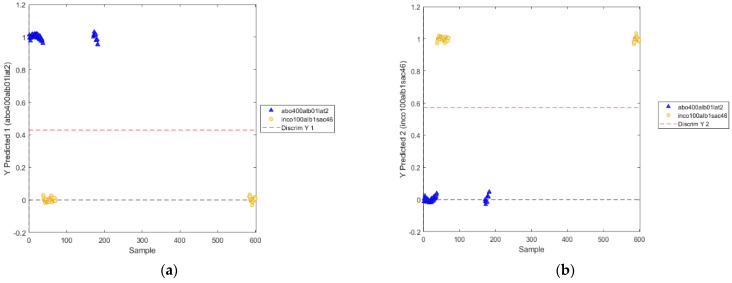
Positions of the discrimination boundary as determined by PLS−DA model (utilized wavelength ranges: 811–991 nm, 1018–1111 nm, 1352–1414 nm, 1527–1722 nm) for the two classes: abobotulinum A toxin (**a**) and incobotulinum A toxin (**b**).

The positions of the discrimination boundary of the novel PLS-DA model for the two classes are shown in Figure 14.

## 4. Discussion

We applied NIR transflectance spectroscopy to analyze the separate solutions of two commercially available formulations of BoNT-A, i.e., abobotulinum A toxin and incobotulinum A toxin.

After preliminary spectra pre-processing (i.e., signal scattering reduction), we applied PCA to discriminate the spectral signatures of the several analyzed solutions and PLS-DA to implement a spectra classification model.

For acquiring transmittance spectra from the solutions, we opted for a transflectance method. Advantages offered by transflectance studies are that sample preparation and spectra acquisition are faster than in transmittance studies—i.e., the solution does not need to be put into a cuvette to acquire the NIR spectrum—and that transflectance performs very well when analyzing both transparent and semi-transparent liquids.

We found that NIR transmittance spectra can discriminate extremely low therapeutically relevant concentrations of distinct formulations of BoNT-A, even when the quantity of excipient proteins overwhelms the toxin itself (e.g., as it is in incobotulinum A toxin formulation) (Figure 2, Figure 5, Figure 7 and Figure 8). In addition, spectra discriminate toxins’ commercial formulations from equiconcentrated solutions of the single excipients (Figure 3, Figure 4 and Figure 6), from saline (Figure 2), and from ultrapure water (Figure 2). Additionally, the separate solutions containing each one of two excipient small sugars—sucrose and lactose—are clearly distinguished (Figure 10). Finally, saline is discriminated from water (Figure 11). Therefore, NIRS proves an especially precise method for identifying components in a solution that represent very low fractions of the total mass of the investigated samples [22].

**Figure 15:**
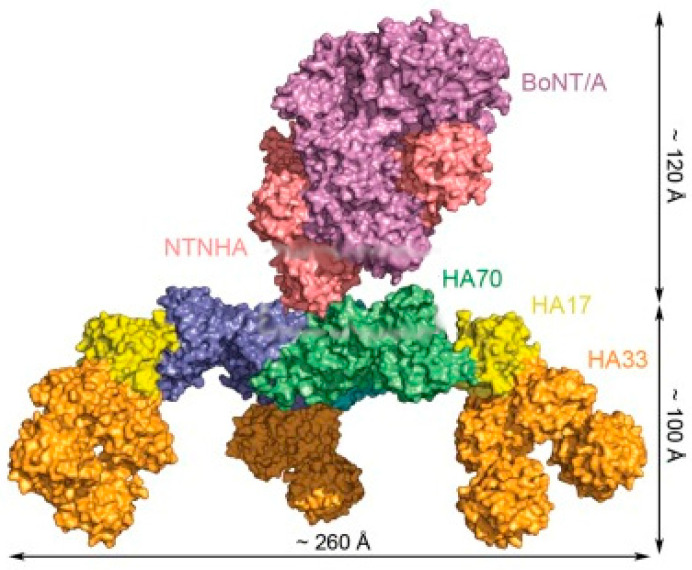
Molecular structure of BoNT-A complex reconstructed by fitting the three-dimensional structures of the BoNT-A and neurotoxin-binding protein (NTNHA) and HA70-HA-17-HA33 complex modules into the electron microscopy image. (Adapted with permission from Ref. [25]. Copyright 2013 Lee et al.).

The main difference between the two studied commercial formulations of botulinum toxin is the presence of complexing proteins in the abobotulinum toxin A vial, whereas incobotulinum A toxin formulation contains the neurotoxin protein alone. In culture supernatants and naturally contaminated foods, botulinum toxin molecules are produced and exist as part of a protein complex termed the progenitor toxin complex (PTC), formed by the association of nontoxic proteins, a nontoxic nonhemagglutinin protein (NTNHA; 130 kDa) and three types of hemagglutinins (HAs) with molecular masses of 70, 33, and 17 kDa (HA-70, HA-33 and HA-17, respectively). Botulinum toxin and NTNHA form heterodimers, which constitute the minimal PTC (m-PTC, ~300 kDa), depicted by its crystal structure as a tight complex resembling interlocked hands. Together with the HA proteins, the heterodimer forms a large triskelion complex [23]. Three HA70s form the central hub; part of HA70 in complex with HA17 and two HA33s form the extended arm. This assembly takes place in steps: first m-PTC is formed, then three HA-70 molecules bind to form L-PTC (large-PTC), and finally, further conjugation of three HA-33/HA-17 trimers (a single HA-17 molecule plus two HA-33 molecules) onto L-PTC/HA-70 follows to produce the mature 750-kDa LL-PTC (extra-large PTC, tetradecameric model or triskelion complex, Figure 15). Therefore, supernatants contain neurotoxin moiety (~150 kDA) and a set of neurotoxic protein complexes, the m-PTC complexes (~300 kDa), the L-PTC complexes (~600 kDa), and the LL-PTC complexes (~900 kDa) [24]. Stabilized through noncovalent interactions, neurotoxin-associated proteins account for up to 70% of the total mass of the complex.

NIR transflectance spectroscopy discriminates the solutions of abobotulinum A toxin from incobotulinum A toxin (Figure 2, Figure 5, Figure 7 and Figure 8) likely due to the different protein content and the specific spatial arrangement of the two proteic solutions. We diluted abobotulinum A toxin at 500 U/1.25 mL to obtain a solution of similar biologic activity as that of incobotulinum A toxin. Units of measurements for these two commercially available BoNT-A preparations are proprietary to each manufacturer and are not interchangeable, but in clinical practice, dose conversion ratios of incobotulinum A to abobotulinum A toxin of 1:4 are reported [26,27]. Since the interactions between the botulinum toxin and neurotoxin-associated proteins are pH dependent (complexing proteins act as a shield for the toxin while crossing the acid environment of the stomach), while it is stable at pH 6.5, the complexes disassembly at pH 7.5. We prevented this event, by diluting the commercial toxin formulations according to the clinical standard, i.e., by using unpreserved 0.9% sodium chloride solution, i.e., not buffered saline. Consistently, pH measurements performed on abobotulinum A toxin and incobotulinum A toxin solutions samples provided acidic values (pH 5.5) for both.

To stabilize the commercial product and to aid in the reconstitution of the neurotoxin from the vial, botulinum toxin formulations contain human serum albumin (HSA) as an excipient [28]. HSA is a highly water-soluble globular monomeric protein that is most abundant in plasma, where it is the main determinant of oncotic pressure and the main modulator of fluid distribution between body compartments [29]. HSA has molecular weight of 67 KDa; it consists of 585 amino acid residues, 1 sulfhydryl group, and 17 disulfide bridges. HSA has an extraordinary ligand binding capacity, providing a depot and carrier for many endogenous and exogenous compounds. The principal binding regions of human serum albumin are in hydrophobic cavities in subdomains IIA and IIIA [30].

NIR transflectance spectroscopy discriminated incobotulinum A toxin from HSA (Figure 2 and Figure 9). Incobotulinum A toxin is the pure neurotoxic protein of BoNT-A. It is a 150-kDa protein, composed of 1260 amino acid residues [31], arranged in a trimodular architecture structured in four domains endowed with different biological properties.

The PCA loadings provide information about the most important wavelength regions in the NIR spectrum that contribute to explain discrimination of the proteins present in the various solutions analyzed (Table 5). Wavelengths associated with water (O-H), non-polar (C-H), polar (S-H), aminic (N-H), amidic (-NR_2_CO) protein residuals were found to be the most important for discrimination both by PCA loadings (Figure 2d,Figure 3d,Figure 4d,Figure 5d,Figure 6d,Figure 7d,Figure 8d and Figure 9d) and VIP scores (Figure 13). Reasonably, differences in the primary, secondary, and tertiary structure between BNT-A, NTNHA protein, the three types of hemagglutinins (HA-70, HA-33 and HA-17), and HSA are the compositional driver of the observed spectral differences in the various analysis performed.

Other excipients used in botulinum neurotoxin commercial formulations include small sugars (sucrose, lactose) and salts (sodium chloride). We acquired spectra from solutions containing each sugar alone, concentrated as in the recommended clinical dilution volumes (Figure 10). Sucrose is a non-reducing disaccharide naturally present in many plants, in varying quantities, with the general formula C_12_H_22_O_11_. It is easily split by hydrolysis into two monosaccharides, i.e., d-glucose (an hexameric ring) and D-fructose (a pentameric ring). Lactose is a reducing sugar primarily found in human and animal milk. It consists of a d-glucose and a d-galactose molecule—both hexameric rings—joined by a β-1,4-glycoside linkage. Lactose has two isomeric forms, α- and β-lactose, which differ with respect to the steric configuration of the hydroxyl group of C-1 moiety of glucose. Small differences in stereochemistry between the different monosaccharides (glucose, fructose, and galactose) determine a significant change in polarity in a solution that is sufficient to influence the sugar−water hydrogen bond interaction and the number of water molecules within the first neighboring shell of the sugar, whether bonded or not [30]. Average hydrogen bond length of sucrose is reported intermediate between that of fructose and glucose, while that of lactose is longer than that of glucose and shorter than that of mannose (fructose < sucrose < glucose < lactose ≪ mannose) [32]. PCA loading plots showed that the structural changes occurring between the small sugars in solutions are detected in two wavelength ranges (1000–1400 nm and 1550–1850 nm, Figure 10d). Interestingly, the second range we found included the band 1742–1746 nm assigned to water shell of glucose anomers in a study aimed at quantifying the anomeric structural changes of glucose solutions using near-infrared spectra [33]. This finding is relevant for our study because the glucose anomer spectra are valuable for interpreting the spectroscopic data of other disaccharides (as those analyzed in the present study) because the bands originating from each glucose anomer appear in the spectra of other carbohydrates. Finally, our findings are in line with previous studies showing that NIRS can discriminate sugars in solution at the millimolar level [34], and it can discriminate sugar and protein from a complex mixture [35].

Transmittance spectra distinguished saline from ultrapure water (Figure 11). Water shows strong absorbance in the mid infrared region but can be measured in NIR, where absorption signals of the various fundamental vibrational bands decrease with orders of magnitude in the first, second, and third overtone regions (800 and 2500 nm). A method exploiting these characteristics is aquaphotomics [36], which relies on the fact that water -OH bonds are altered easily by other molecules, thus giving the opportunity to investigate the changes induced by the solute in the water molecular system itself. By evaluating the absorption bands related to the overtones and combinations of stretching and bending vibrations of -OH, NIR describes the structural changes, interactions, and conformations within the liquid water, including all molecular vibrations at specific water wavelength bands induced by the solute changes described [37]. Our findings showing that saline is distinguished from water add to many other reports showing that signals of low-concentration compounds are best detectable with NIR compared to the fundamental region, owing to the use of water absorbance bands [38]. PCA loadings constantly show 800–1100 nm, 1450–1750 nm, and 1900–2200 nm regions (Figure 11d), which coincide with huge water absorbance bands—second overtone of water, first overtone of water and combination band. Because salts are practically transparent for NIR light, our results depend entirely on the changes in the water molecular matrix [39], thereby perfectly illustrating the aquaphotomics water-molecular and energy mirror concept [40]. Because the instrumentations and measurements in the NIR region are cost effective, the present findings provide both practical and theoretical benefit to investigators aiming to analyze elements in highly diluted systems [34].

Possible effects of refractive index and scattering probability of the samples on the discrimination deserve a final comment. It cannot be excluded that differences in the raw transmittance between 800 and 1200 nm may originate, at least in part, from factors such as baseline drift due to reflection between the collimation lens and samples or Rayleigh scattering rather than the overtone absorption. However, to minimize—and possibly prevent—such factors, in our experiments, we maintained consistent the set-up configuration (i.e., the angle of incident light and the distance of light illumination/collection) through all the measurements; we connected an attenuator to light source and fiber optic cable to optimize the baseline; we updated the dark current frequently during spectra collection (to minimize the effect of the drift); and we measured the baseline before collecting spectra from each sample. Moreover, the spectrophotometer averages ten raw spectra for each one of the fifty spectra collected, and noise is known to decrease at the square root of the number of scans averaged. Obviously, nothing can be done to solve the spectral sensitivity drift due to the jumps around the ASD field spectrophotometers detector’s gaps, which, by manufacturer’s design, are at wavelength 1001 nm and 1801 nm.

## 5. Conclusions and Future Perspectives

This proof-of-concept study shows that NIR transflectance spectroscopy distinguishes distinct solutions of commercial formulations of botulinum neurotoxin type A diluted at standard clinical concentrations (abobotulinum A toxin vs. incobotulinum A toxin), distinct proteins (HSA vs. neurotoxin vs. complexing proteins), very diluted solutions of small sugars (lactose vs. sucrose), and saline from water. Principal component analysis proved to be a good technique to explore the spectral features of the two BoNT-A solutions and those of the various excipients diluted according to clinical standards. The set-up classification models were able to discriminate the two analyzed BoNT-A solutions with the highest precision and accuracy values.

## Figures and Tables

**Table 1:** Sample composition.

Sample ID	Composition	Temperature (°C)	pH
NC09	Saline solution (NaCl 0.9%)	18	5.5
alb1	1 mg of albumin in 1 mL NaCl 0.9%	18	5.5
sac46	4.6 mg of sucrose in 1 mL NaCl 0.9%	18	5.5
lact1	1 mg of lactose in 1 mL NaCl 0.9%	18	5.5
AD	Ultrapure Water	18	5.8
abo400alb01lat2	500 U/1.25 mL abobotulinum A toxin (**)	18	5.5
abo100alb0025lat05	100 U/mL abobotulinum A toxin (*)	18	5.5
inco100alb1sac46	100/1 mL incobotulinum A (*)	18	6.0
EccAbo	0.125 mg albumine; 2.5 lactose; saline (NaCl 0.9%) 2.5 mL	18	5.5
Eccinco1	1 mg albumine, 4.6 mg sucrose; saline (NaCl 0.9%) 1 mL	18	5.5

(*) clinical diluition. (**) equiactivity.

**Table 2:** Comparison of the two commercial formulations of abobotulinum A and incobotulinum A toxin.

Botulinum Toxin Type A	Abobotulinumtoxin A	Incobotulinumtoxin A
Presentation	Freeze-died (lyophilized) powder for reconstitution	Freeze-died (lyophilized) powder for reconstitution
Isolation process	Precipitation and chromatography	Precipitation and chromatography
Composition	Clostridium botulinum toxin type A; hemagglutinin (HA) and non-HA proteins	Clostridium botulinum toxin type A
Excipients^a^	500 U vial: human serum albumin 125 µg; lactose 2.5 mg	500 U vial: human serum albumin 1 mg; sucrose 4.6 mg
Molecular weight (neurotoxin, kDa)	Not published (150)	150
Approximate total clostridial protein content (ng per 100 U)	4.87	0.44
Neurotoxin protein load (ng neurotoxin potency per 100 U ^a^)	0.65	0.44
Specific neurotoxin potency (U/ng)	154	227

^a^ Units of measurements for these two commercially available BoNT/A preparations are proprietary to each manufacturer and are not interchangeable.

**Table 3:** PLS-DA performance metrics for discriminating incobotulinum A toxin (inco100alb1sac46) and abobotulinum A toxin (abo400alb01lat2), using the wavelength range 800–2400 nm.

	Class	Sensitivity	Specificity	Number of Spectra	Error	Precision	Accuracy
**Calibration**	abo400alb01lat2	1.000	1.000	37	0.000	1.000	1.000
	inco100alb1sac46	1.000	1.000	33	0.000	1.000	1.000
**Cross-validation**	abo400alb01lat2	1.000	1.000	37	0.000	1.000	1.000
	inco100alb1sac46	1.000	1.000	33	0.000	1.000	1.000
**Prediction**	abo400alb01lat2	1.000	1.000	13	0.000	1.000	1.000
	inco100alb1sac46	1.000	1.000	17	0.000	1.000	1.000

**Table 4:** Performance metrics for PLS-DA of incobotulinum A toxin (inco100alb1sac46) and abobotulinum A toxin (abo400alb01lat2), using the wavelength ranges selected by the VIP scores method: 811–991 nm, 1018–1111 nm, 1352–1414 nm, 1527–1722 nm.

	Class	Sensitivity	Specificity	Number of Spectra	Error	Precision	Accuracy
**Calibration**	abo400alb01lat2	1.000	1.000	37	0.000	1.000	1.000
	inco100alb1sac46	1.000	1.000	33	0.000	1.000	1.000
**Cross-validation**	abo400alb01lat2	1.000	1.000	37	0.000	1.000	1.000
	inco100alb1sac46	1.000	1.000	33	0.000	1.000	1.000
**Prediction**	abo400alb01lat2	1.000	1.000	13	0.000	1.000	1.000
	inco100alb1sac46	1.000	1.000	17	0.000	1.000	1.000

**Table 5:** Biochemical features of the proteins diluted in the various solutions analyzed.

	BoNT-A	NTNHA Type A	HA-70 Type C	HA-33 Type C	HA-17 Type D	HSA
**-OH**	145	234	150	66	41	48
**-COOH**	165	145	62	23	11	98
**-NH2** **-NR2CO**	323	304	156	93	35	140
**-SH**	9	12	4	3	1	35
**H-Bonds**	1.926	2.085	1.116	555	264	963
**M.W.**	149 kDa	138 kDa	70.6 K kDa	33.7 K kDa	16.7 K kDa	69 kDa
**I.p.**	5.50	4.89	5.18	8.25	5.23	5.92

-OH, -COOH, -NH2, -NR2CO, -SH =, respectively: hydroxyl group, carboxylic group, amino group, group amide, thiol group; H-Bonds = estimation of hydrogen interactions by the whole protein; M.W. = Molecular weight; I.p. = Isoelectric point. (Data from National Center for Biotechnology Information, https://www.ncbi.nlm.nih.gov/protein/P0DPI1.1 (accessed on 16 March 2022); https://www.ncbi.nlm.nih.gov/protein/P0DPI1.1?report=fasta (accessed on 17 March 2022); I.p. and M.W. calculation: Swiss Bioinformatics Resource Portal Expasy; https://web.expasy.org/compute_pi/( accessed on 16 March 2022); Protein composition calculation; NMR Groups in the Laboratory of Chemical Physics https://spin.niddk.nih.gov/clore/Software/A205.html (accessed on 17 March 2022).

## Data Availability

The datasets generated during and/or analyzed during the study are available from the corresponding author on reasonable request.
